# Evaluation of symptomatic slow-acting drugs in osteoarthritis using the GRADE system

**DOI:** 10.1186/1471-2474-9-165

**Published:** 2008-12-16

**Authors:** Olivier Bruyère, Nansa Burlet, Pierre D Delmas, René Rizzoli, Cyrus Cooper, Jean-Yves Reginster

**Affiliations:** 1 WHO Collaborating Center for the Public Health Aspect of Musculoskeletal Disorders, University of Liege, Belgium; 2 International Osteoporosis Foundation, Nyon, Switzerland; 3 INSERM 831 Research Unit, Lyon, France; 4 Department of Rehabilitation and Geriatrics, Geneve, Switzerland; 5 MRC Epidemiology Resource Center, Southampton, UK

## Abstract

**Background:**

Symptomatic slow-acting drugs (SYSADOA) have been largely studied over the last decade. The objective of this study is to prepare a document providing recommendations for the use of SYSADOA in osteoarthritis (OA).

**Methods:**

The following interventions were taken into consideration: avocado/soybean unsaponifiables, chondroitin sulfate, diacereine, glucosamine sulfate, hyaluronic acid, oral calcitonin, risedronate, strontium ranelate. Recommendations were based on the GRADE (Grading of Recommendations Assessment, Development and Evaluation) system. The GRADE system is based on a sequential assessment of the quality of evidence, followed by assessment of the balance between benefits versus downsides and subsequent judgment about the strength of recommendations.

**Results:**

Chondroitin sulfate, diacereine, glucosamine sulfate, avocado/soybean unsaponifiables and hyaluronic acid have demonstrated pain reduction and physical function improvement with very low toxicity, with moderate to high quality evidence. Even if pre-clinical data and some preliminary in vivo studies have suggested that oral calcitonin and strontium ranelate could be of potential interest in OA, additional well-designed studies are needed.

**Conclusion:**

In the benefit/risk ratio, the use of chondroitin sulfate, diacereine, glucosamine sulfate, avocado/soybean unsaponifiables and hyaluronic acid could be of potential interest for the symptomatic management of OA.

## Background

Osteoarthritis (OA) is a progressive disorder characterized by destruction of articular cartilage and subchondral bone associated with synovial changes [1,2]. This degenerative condition affects aging men and women [3]. The two most affected location for pain and physical disability in adults are hip and knee [4]. Because of its important prevalence worldwide, OA represents a huge burden in terms of individual, as well as public health resources utilization [5]. Pharmacological and non-pharmacological procedures have demonstrated their efficacy to stop or decrease progression of this condition. Among pharmacological treatments, symptomatic slow-acting drugs have been largely studied over the last decade.

Most countries face common challenges in delivering consistent, appropriate and high quality health care standards within the limits of available resources. Clinical guidelines are one of the most important options to support and promote good clinical practices, and subsequently to make patient care more effective and efficient. However, to ensure that clinical guidelines actually meet this objective, they should follow a strict, validated methodology.

Over the last decade, several scientific societies involved in OA produced guidelines for the management of hip, knee and hand to improve quality and effectiveness of patients care [6-11]. Nevertheless, the most recent versions which have been prepared by prestigious institutions, such as the *American College of Rheumatology *(ACR) or the *European League Against Rheumat*ism (EULAR) do not include the latest original research papers [12-28]. Furthermore, the recommendations established on the same topic by these two groups often differ. Part of the reasons are the lack of evidence, different interpretation of evidence, unsystematic guideline development methods, influence of professional bodies, cultural and socio-economic factors and differences in health care systems [29-31].

For all these reasons the *European Society for Clinical and Economic Aspects of Osteoporosis and Osteoarthritis *(ESCEO) experts found appropriate to build their own recommendations for the use of symptomatic slow-acting drugs in osteoarthritis (SYSADOA) based on the GRADE system [32,33]. The acronym of GRADE stands for Grading of Recommendations Assessment, Development and Evaluation.

The GRADE system is based on a sequential assessment of the quality of evidence, followed by assessment of the balance between benefits versus downsides and subsequent judgment about the strength of recommendations. Because frontline consumers of recommendations will be most interested in the best course of action, the GRADE system places the strength of the recommendation first, followed by the quality of the evidence. Separating the judgments regarding the quality of evidence from judgments about the strength of recommendations is a critical and specific feature of this new grading system.

GRADE has two level of recommendation: strong and weak (Table 1). As a matter of fact, recommendations to administer, or not administer, an intervention, should be based on trade-offs between benefits and risks, burden and, where possible, costs. If benefits outweigh risks and burden, experts will recommend that clinicians offer a treatment to patients sustaining typical symptoms of the disease. The uncertainty associated with the trade-off between the benefits and risks and burdens will determine the strength of recommendations.

**Table 1:** Strength of guideline recommendations, consensus-based statements, and implication to quality of evidence

**Recommendation or statement**	**Description in GRADE approach**	**Interpretation**
Strong guideline recommendation	We recommend (should)	1. Most individuals should receive the intervention, assuming that they have been informed about and have understood its benefits, harms and burden.
		2. Most individuals would want the recommended course of action and only a small proportion would not.
		3. The recommendation could unequivocally be used for policy making.

Weak guideline recommendation	We suggest (might)	1. The majority of individuals would want to suggested course of action, but an appreciable proportion would not.
		2. Values and preferences vary widely.
		3. Policy making will require extensive debates and involvement of many stakeholders.

## Methods

A systematic and exhaustive search of the meta-analysis and randomized controlled trials published from 1950 until December 2007 has been undertaken, using several tools, such as Medline, Old Medline, Embase, CINAHL, Science Citation Index through Web of Science, Allied Complementary Medicine and Cochrane Library databases. The search in the Cochrane Library included the Cochrane Reviews, Abstracts of Quality Assessed Systematic Reviews, The Cochrane Controlled Trial Register, NHS Economic Evaluation Database, Health Technology Assessment Database and NHS Economic Evaluation Bibliography Details Only.

Quality of the evidence has been assessed using the grade four-category system (high, moderate, low and very low quality) (Table 2).

**Table 2:** Quality of the quality evidence, definitions and underlying methodology

**Grade**	**Definition**	**Underlying Methodology**
High	Further research is very unlikely to change our confidence in the estimate of effect	RCT or meta-analysis
Moderate	Further research is likely to have an important impact on our confidence in the estimate of effect and may change the estimate	Downgraded RCTs or upgraded observational studies
Low	Further research is very likely to have an important impact on our confidence in the estimate of effect an its likely to change the estimate	Well-done observational studies with control groups
Very low	Any estimate of effect is very uncertain	Others (e.g., case reports or case series)

Factors that are considered in classifying evidence are: the study design and rigour of its execution, the consistency of results and how well the evidence can be directly applied to patients, interventions, outcomes and comparator. Other important factors are whether the data are sparse or imprecise and whether there is potential for reporting bias. Using this approach, assessments of the quality of evidence for each important outcome take into account the study design, limitations of the studies, consistency of the evidence across studies, the directness of the evidence, and the precision of the estimate.

Obviously, all outcomes (e.g. pain, function, NSAIDS consumption, carry-over effect, harm, global patient satisfaction, use of walking aids, structure modification, and evaluation by the GP) have not the same importance. The importance of each outcome has then been scored (10 mm VAS) independently by each expert. Only outcomes considered as important (VAS > 6 mm) by all experts were discussed in these recommendations.

For each intervention considered, the panel formulated a consensus recommendation based on the panel members' judgments regarding the balance between the benefits, harms (adverse effects), burdens (e.g., taking medication daily), costs, and values and preferences (the desirability or preference that individuals exhibit for a particular outcome) of the intervention. Source of funding has not been considered. Then, recommendations have been classified as "strong" or "weak."

## Results

The experts considered that pain, function and harm are of primary interest in the evaluation of SYSADOA.

The following interventions were taken into consideration:

- Avocado/soybean unsaponifiables [34-36].

- Chondroitin sulfate [37-40].

- Diacereine [41,42].

- Glucosamine sulfate [38,39,43-45].

- Hyaluronic acid [46-52].

- Oral calcitonin [53].

- Risedronate [54,55].

- Strontium ranelate [56].

Following the GRADE system, the study design for all trials included in the review of evidence for chondroitin sulfate, diacereine, glucosamine sulfate, hyaluronic acid and risedronate was randomised controlled trial which is scored as a high type of evidence. As requested from the methodology of GRADE, study quality was also assessed by reviewing whether the studies had limitations or flaws. The following limitations were noted, leading frequently to a decrease in the quality of evidence: methods of randomisation were not clearly reported, allocation concealment was not reported or unclear, some trials were single-blinded and frequently the method of blinding was not reported in detail, incomplete descriptions of withdrawals and dropouts were reported, analyses were based on the per protocol or completer population and not on the intention-to-treat population, heterogeneity not tested in meta-analysis, large heterogeneity between meta-analyses statistically significant differences were reported at baseline between treatment and control groups, different severity of knee OA patients were included, large results discrepancies (e.g. between studies or between meta-analysis and large recent studies), nature of the placebo comparator, very few number of patient included, post-hoc analysis.

Quality evidence could also decrease when considering meta-analysis (e.g. moderate and high heterogeneity between RCT, or heterogeneity between meta-analyses).

The summary of the grading recommendation is summarized in table 3 and the text below summarizes the most important data for each compound.

**Table 3:** Recommendations taking into account the balance of benefit (pain reduction and function improvement) and harm (adverse event)

**Product**	**Grade of recommendation**	**Quality evidence**	**Balance benefit to harm**
*Avocado/soybean unsaponifiables*	Strong	Moderate	Avocado/soybean unsaponifiables advantageous
*Chondroitin sulfate*	Strong	Moderate	Chondroitin sulfate advantageous
*Diacereine*	Strong	Moderate	Diacereine advantageous
*Glucosamine sulfate*	Strong	Moderate	Glucosamine sulfate advantageous
*Hyaluronic acid*	Strong	Moderate	Hyaluronic acid advantageous
*Oral calcitonin*	Weak	Low	Calcitonin not advantageous
*Risedronate*	Strong	High	Risedronate not advantageous
*Strontium ranelate*	Weak	Very low	Strontium ranelate advantageous

### 1. Avocado/soybean unsaponifiables

Three RCTs have been selected for our recommendations.

The first one is a 3-month, prospective, randomized, double-blind, placebo-controlled trial evaluating the efficacy of avocado/soybean unsaponifiables (300 mg/day) in terms of NSAID use reduction [34]. Secondary efficacy criteria were the visual analogic scale pain score and the Lequesne index. After 3 months, the functional index showed a significantly greater improvement in the active drug group (-2.3 +/- 2.6) than in the placebo group (-1.0 +/- 2.6) (p < 0.01). However, pain scores over time were similar in the two groups.

The second study (n = 260), of the same duration, showed that avocado/soybean unsaponifiables (300 or 600 mg/day) significantly reduced the Lequesne index (secondary outcome) compared to placebo [35]. After 6 months of follow-up, the mean (SD) Lequesne score decreased from 9.6 +/- 2.5 to 5.5 +/- 3.6 in the avocado/soybean unsaponifiables 300 mg/day, from 9.8 +/- 2.7 to 6.5 +/- 3.5 in the avocado/soybean unsaponifiables 600 mg/day and from 9.8 +/- 2.4 to 7.8 +/- 3.4 in the placebo group (p < 0.01 between placebo and the two avocado/soybean unsaponifiables groups).

The third RCT included 164 patients with primary OA of the knee (n = 114) or hip (n = 50) with a 6-month treatment period and a 2-month post-treatment follow-up [36]. The results showed that avocado/soybean unsaponifiables (300 or 600 mg/day) significantly reduced the Lequesne index (primary outcome) and the pain score (VAS scale) compared to placebo. After 6 months of follow-up, the mean (SEM) Lequesne score decreased from 9.7 +/- 0.3 to 6.8 +/- 0.4 in the avocado/soybean unsaponifiables and from 9.4 +/- 0.3 to 8.9 +/- 0.4 in the placebo group (p < 0.001). Pain decreased from 56.1 +/- 1.6 mm to 35.3 +/- 2.3 in the avocado/soybean unsaponifiables and from 56.1 +/- 1.8 to 45.7 +/- 2.6 in the placebo group (p = 0.003).

In these 3 trials, avocado/soybean unsaponifiables were well tolerated.

### 2. Chondroitin sulfate

Four meta-analyses have been selected. However, two of them, showing a significant effect of chondroitin sulfate compared to placebo, were outdated since they were issued before the appearance of major recent trials [37,38]. One of them is also difficult to use since the authors evaluated chondroitin sulfate together with glucosamine sulfate [39].

In the last meta-analysis, 20 trials (3846 patients) were included [40]. The meta-analysis identified a significant beneficial effect of chondroitin sulfate on pain, with an effect size of -0.75 (-0.99 to -0.50). However, the heterogeneity between trials was high (I^2 ^= 92%). When the authors restricted the analysis to the 3 trials with large sample sizes and an intention-to-treat analysis, the effect size was -0.03 (-0.13 to 0.07; I^2 ^= 0%) and corresponded to a difference of 0.6 mm on a 10-cm visual analogic scale. However, this restricted analysis included one study with an exceptionally high placebo response rate, one study that was only published as an abstract.

At last, a meta-analysis of 12 trials showed a pooled relative risk of 0.99 (0.76 to 1.31) for any adverse event between chondroitin sulfate and placebo [40].

### 3. Diacerein

Two meta-analyses have been included.

The fist one included 19 studies (search date 1985–2004) [41]. Diacerein was significantly superior to placebo to reduce pain and improve function during the active treatment phase (Glass score 1.50 [0.80–2.20]). Moreover, diacerein showed a carryover effect, persisting up to 3 months after treatment, with a significant analgesic-sparing effect during the follow-up period (Glass score 2.06 [0.66 to 3.46]). Nevertheless, it should be pointed out that heterogeneity was not tested in this meta-analysis.

The second one included 7 studies (search date 1966–2004) with 2069 participants [42]. The results of the meta-analysis showed a small, consistent, beneficial effect of diacerein in the treatment of OA. When compared to placebo, pain on a visual analogic scale (0–100 mm) showed a statistically significant difference in favour of diacerein (weighted mean differences of -5.16 [-9.75 to -0.57]); but the heterogeneity analysis result was important (p = 0.04). No significant effect of diacereine was observed on the Lequesne index for function compared to placebo, with homogeneity in all results. The most frequent adverse event was diarrhoea. 459 participants among 1083 participants that received diacerein (42%) were affected. 18% in the treatment group compared with 13% in the placebo group withdrew due to adverse events.

### 4. Glucosamine sulfate

Five meta-analysis and systematic reviews were included. Three groups of systematic reviews and meta-analyses emerged:

▪ A Cochrane Review, first published in 2001 and updated in 2005 [43], that includes every relevant glucosamine trials but studies published in 2006 and 2007. This meta-analysis remains the only one that includes all reference (NSAIDs)-controlled trials. In addition, it provides an evaluation on safety aspects.

▪ Meta-analyses by a group in Boston. The first was published in 2000 and is clearly outdated since it was issued before the appearance of all recent and most relevant trials [38]. However, Vlad et al. updated their meta-analysis in 2007 and this included all relevant placebo-controlled glucosamine trials [44].

▪ Systematic reviews and meta-analyses performed by the group in Liege. The first was published in 2003 and had the merit to be the first to include the glucosamine sulfate long-term trials [39]. However, aside not being up to date, it is also difficult to use for the assessment of glucosamine sulfate studies on the symptoms of OA, since these trials are evalutated together with those of chondroitin sulfate. Nevertheless, Reginster updated this group's systematic review and meta-analysis in 2007 in an editorial of the meta-analysis by Vlad et al., using in his approach the pivotal trials of prescription glucosamine sulfate [44,45].

Three systematic reviews and meta-analyses have been used in the present assessment [43-45].

In the Cochrane review, the 20 analyzed RCTs found glucosamine favoured placebo with a 28% (change from baseline) improvement in pain (standardized mean difference of -0.61 [-0.95 to -0.28] and a 21% (change from baseline) improvement in function using the Lequesne index (standardized mean difference of -0.51 [-0.96 to -0.05]) [43].

Vlad et al. also reported a significant effect of glucosamine sulfate for pain improvement (effect size of 0.35 [0.14 to 0.56]) [44]. However, heterogeneity was high among the 15 RCTs included in their meta-analysis (I^2 ^= 80%).

Reginster based his meta-analysis only on 3 specific pivotal trials of glucosamine sulfate [45]. Pivotal trials are high-quality studies used by Health Authorities to assess the efficacy and safety of a prescribed medication in order to grant the marketing authorisation. The assessments of WOMAC pain and WOMAC function provide a significant beneficial effect of glucosamine sulfate compared to placebo (effect size of 0.27 [0.12 to 0.43]) and 0.33 [0.17–0.48], respectively), without heterogeneity (I^2 ^= 0%).

At least, glucosamine sulfate was as safe as placebo, in terms of subjects reporting adverse reactions (RR = 0.97 [0.88 to 1.08]) [43].

### 5. Hyaluronic Acid

Seven systematic reviews have been included in this assessment.

Analysis of these meta-analyses showed discrepancies between systematic reviews/meta-analyses of the efficacy and safety of hyaluronic acid (HA) therapy in the treatment of osteoarthritis [46-52].

Out of the 11 RCTs included in a meta-analysis, it has been demonstrated that the 100-mm visual analogic scale differences between therapy and placebo injection was 4.4 (1.1 to 7.2) at 1 week, 17.7 (7.5 to 28.0) at 5 to 7 weeks, 18.1 (6.3 to 29.9) at 8 to 12 weeks, and 4.4 (-15.3 to 24.1) at 15 to 22 weeks [51]. Another meta-analysis, including 22 RCTs, showed that patients who received the intervention treatment experienced a reduction in pain during movement: the mean difference on a 100-mm visual analogue scale was -3.8 mm (-9.1 to 1.4 mm) after 2–6 weeks, -4.3 mm (-7.6 to -0.9) after 10–14 weeks and -7.1 mm (-11.8 to -2.4) after 22–30 weeks [47]. However, this effect was not considered as being clinically meaningful [47]. Another meta-analysis showed that, from the 22 studies included, the pooled effect size for hyaluronic acid was 0.32 (0.17 to 0.47) when considering pain reduction but with a significant heterogeneity among studies (P < .001) [48].

Interestingly, the reasons for inconsistency between systematic reviews have been recently searched by J. Campbell et al. [57]. They identified inclusion of different controlled trials as a result of different search strategies and selection criteria, differences in the outcome measures and time points selected for extraction; and different statistical methods for data synthesis, which resulted in conflicting estimates of therapeutic effect. Anyway, the authors concluded that although the overall quality was moderate, there were net benefits (pain reduction and physical function) in favour of HA compared to placebo with low risk of harm.

### 6. Oral calcitonin

One RCT has been included. In this small randomized, double-blind trial, patients received either placebo (n = 18), 0.5 mg of oral salmon calcitonin (n = 17), or 1 mg of oral salmon calcitonin (n = 18) daily for 84 days [53]. No significant improvements were observed at the end of the study between patients on placebo or oral calcitonin, in the Lequesne index (secondary outcome).

### 7. Risedronate

Two RCTs have been included in this analysis.

The first trial included 2483 patients (placebo, 5 or 15 mg risedronate) in a 2-year study [54]. No significant effect of risedronate has been observed in the WOMAC score, compared to placebo. No increase in the number of adverse events was demonstrated for risedronate compared with placebo.

In the second study, 285 patients were randomized to once-daily risedronate (5 mg or 15 mg) or placebo, in a 1-year prospective, double-blind, placebo-controlled trial [55]. Those receiving risedronate showed no improvement of the WOMAC index, compared to placebo. Both doses of risedronate were well tolerated.

### 8. Strontium ranelate

One RCT has been included.

This RCT is a post-hoc analysis of two trials aiming at assessing the efficacy and safety of strontium ranelate in the treatment of postmenopausal osteoporosis [56]. The results showed that among 399 osteoporotic women with concomitant radiological spinal OA, significantly more patients in the strontium ranelate group experienced an improvement in back pain after 3 years (secondary analysis), compared with placebo (p = 0.03).

## Discussion

In the light of these results, some SYSADOA have a positive risk benefit balance for patients with OA. As a matter of fact, chondroitin sulfate, diacereine, glucosamine sulfate, avocado/soybean unsaponifiables and hyaluronic acid have demonstrated pain reduction and physical function improvement with very low toxicity, with moderate to high quality evidence. The only treatment with a substantial highest level of adverse events was diacerheine. The most frequent adverse effect was mild to moderate diarrhoea, which usually appeared at an early stage during treatment and resolved on continuing treatment. However, this adverse event did not result in treatment interruption in the majority of the patients.

Based on our research, there are only two treatments that are "weakly" recommended. Indeed, even if pre-clinical data and some preliminary in vivo studies have suggested that oral calcitonin and strontium ranelate could be of potential interest in OA, additional well-designed studies are needed.

It should be pointed out that some meta-analysis, even with positive results for the treatment compared to placebo, have strong evidence of heterogeneity and, with consequences, a lowest quality evidence for the treatment, following the GRADE recommendation (e.g. reduced from "high" to "moderate").

Results of the GAIT trial are also of interest and deserve special comments as this study involves glucosamine and chondroitin [58]. This National Institutes of Heath sponsored study examined placebo versus glucosamine hydrochloride (500 mg three times daily) versus chondroitin sulfate (400 mg three times daily) versus the combination of glucosamine and chondroitin versus celecoxib (200 mg/day) in a parallel, blinded 6 month multicenter study of response in knee OA. The primary efficacy variable was a 20% improvement in knee pain from baseline to 24 weeks. Overall, glucosamine and chondroitin sulfate were not significantly better than placebo in reducing knee pain by 20 percent. However, for patients with moderate-to-severe pain at baseline, the rate of response (OMERACT-OARSI criteria) was significantly higher with combined therapy than with placebo (79.2% vs. 54.3%, P = 0.002). The high placebo response (60.1%) is of unknown significance but might explain the findings of the GAIT trial. As a matter of fact, if placebo is effective in 60 percent of patients, it could be difficult for other treatments to surpass this mark. At least, this study used the glucosamine hydrochloride 500 mg three times daily comparted to glucosamine sulfate 1500 once daily in the most positive trials,

It should be acknowledged, however, that the size effect in pain and physical improvement is only considered from small to moderate. At least, the duration of the RCT differs widely (3 months to 3 years), within and between treatments, making the interpretation sometime more difficult.

It should also be pointed out that some potential treatments have only been assessed in one site and that, partly because of the difference in the physiopathology between hip and knee osteoarthritis, results obtained at the level of the knee can not be extrapolated to the hip. For example, it has been shown in one study that glucosamine sulfate appears to be ineffective in hip osteoarthritis [59].

At least, although we have used the GRADE approach to rate the quality of evidence and strength of recommendation, the need for judgment is still required. Indeed, RCTs or meta-analysis of the same product could have important methodological differences that may impact on the results. At least, even if the use of risk/benefit ratio is of great potential interest, it still needs further validation. It should also be pointed out, as a limitation of this work, that studies were not blinded and, consequently, some experts reviewed the quality of their own works.

## Conclusion

In conclusion, in the benefit/risk ratio, the use of chondroitin sulfate, diacereine, glucosamine sulfate, avocado/soybean unsaponifiables and hyaluronic acid could be of potential interest for the symptomatic management of OA.

## Competing interests

This article is based on the outcomes of a Working Group meeting convened by ESCEO. Some of the authors on this article have received support from, or, have given talks, attended conferences and participated in trials and advisory boards sponsored by various pharmaceutical companies. OB has received consulting fees or has been reimbursed for attending scientific meetings from Theramex, Rotta, GlaxoSmithKline, Servier, Galapados. PDD has received consulting fees, grant or has participated in paid advisory boards from Acceleron, Amgen, Eli Lilly, GSK, MSD, Novartis, Nycomed, Organon, Pfizer, Procter & Gamble, Roche, Sanofi-Aventis, Servier, Wyeth, Zelos. RR has received consulting fees, lectures fees, grant or has participated in paid advisory boards from Servier, Novartis, Amgen, GlaxoSmithKline, Roche, Nycomed, Procter & Gamble, Merck Sharp and Dohme, Lilly. CC has received consulting fees, lectures fees, grant or has participated in paid advisory boards from Servier, Proctor & Gamble/Alliance for better bone health, Merck Sharp & Dohme, Eli Lilly, GSK/Roche. JYR has received consulting fees, lectures fees, grant or has participated in paid advisory boards from Servier, Novartis, Negma, Lilly, Wyeth, Amgen, GlaxoSmithKline, Roche, Merckle, Nycomed, NPS, Merck Sharp and Dohme, Theramex, Rottapharm, IBSA, Genevrier, Teijin, Teva, Ebewee Pharma, Zodiac, Analis, Novo-Nordisk, Bristol Myers Squibb. However, the authors confirm that no pharmaceutical company has been involved with the drafting and publication of this article, financially or otherwise.

## Authors' contributions

All authors have participated in the working group meeting. OB drafted the manuscript. All authors reviewed and approved the manuscript.

## Pre-publication history

The pre-publication history for this paper can be accessed here:



## References

[B1] Goldring SR, Goldring MB (2006). Clinical aspects, pathology and pathophysiology of osteoarthritis. J Musculoskelet Neuronal Interact.

[B2] Martel-Pelletier J, Lajeunesse D, Fahmi H, Tardif G, Pelletier JP (2006). New thoughts on the pathophysiology of osteoarthritis: one more step toward new therapeutic targets. Curr Rheumatol Rep.

[B3] Garstang SV, Stitik TP (2006). Osteoarthritis: epidemiology, risk factors, and pathophysiology. Am J Phys Med Rehabil.

[B4] Arden N, Nevitt MC (2006). Osteoarthritis: epidemiology. Best Pract Res Clin Rheumatol.

[B5] Jinks C, Jordan K, Croft P (2007). Osteoarthritis as a public health problem: the impact of developing knee pain on physical function in adults living in the community: (KNEST 3). Rheumatology (Oxford).

[B6] Zhang W, Moskowitz RW, Nuki G, Abramson S, Altman RD, Arden N (2007). OARSI recommendations for the management of hip and knee osteoarthritis, Part I: Critical appraisal of existing treatment guidelines and systematic review of current research evidence. Osteoarthritis Cartilage.

[B7] (2000). Recommendations for the medical management of osteoarthritis of the hip and knee: 2000 update. American College of Rheumatology Subcommittee on Osteoarthritis Guidelines. Arthritis Rheum.

[B8] Jordan KM, Arden NK, Doherty M, Bannwarth B, Bijlsma JW, Dieppe P (2003). EULAR Recommendations 2003: an evidence based approach to the management of knee osteoarthritis: Report of a Task Force of the Standing Committee for International Clinical Studies Including Therapeutic Trials (ESCISIT). Ann Rheum Dis.

[B9] Zhang W, Doherty M, Arden N, Bannwarth B, Bijlsma J, Gunther KP (2005). EULAR evidence based recommendations for the management of hip osteoarthritis: report of a task force of the EULAR Standing Committee for International Clinical Studies Including Therapeutics (ESCISIT). Ann Rheum Dis.

[B10] Zhang W, Doherty M, Leeb BF, Alekseeva L, Arden NK, Bijlsma JW (2007). EULAR evidence based recommendations for the management of hand osteoarthritis: report of a Task Force of the EULAR Standing Committee for International Clinical Studies Including Therapeutics (ESCISIT). Ann Rheum Dis.

[B11] Zhang W, Moskowitz RW, Nuki G, Abramson S, Altman RD, Arden N (2008). OARSI recommendations for the management of hip and knee osteoarthritis, Part II: OARSI evidence-based, expert consensus guidelines. Osteoarthritis Cartilage.

[B12] Altman RD, Zinsenheim JR, Temple AR, Schweinle JE (2007). Three-month efficacy and safety of acetaminophen extended-release for osteoarthritis pain of the hip or knee: a randomized, double-blind, placebo-controlled study. Osteoarthritis Cartilage.

[B13] Avouac J, Gossec L, Dougados M (2007). Efficacy and safety of opioids for osteoarthritis: a meta-analysis of randomized controlled trials. Osteoarthritis Cartilage.

[B14] Baker K, Goggins J, Xie H, Szumowski K, LaValley M, Hunter DJ (2007). A randomized crossover trial of a wedged insole for treatment of knee osteoarthritis. Arthritis Rheum.

[B15] Batlle-Gualda E, Roman Ivorra J, Martin-Mola E, Carbonell Abello J, Linares Ferrando LF, Tornero Molina J (2007). Aceclofenac vs paracetamol in the management of symptomatic osteoarthritis of the knee: a double-blind 6-week randomized controlled trial(1,2). Osteoarthritis Cartilage.

[B16] Brismee JM, Paige RL, Chyu MC, Boatright JD, Hagar JM, McCaleb JA (2007). Group and home-based tai chi in elderly subjects with knee osteoarthritis: a randomized controlled trial. Clin Rehabil.

[B17] Cantarini L, Leo G, Giannitti C, Cevenini G, Barberini P, Fioravanti A (2007). Therapeutic effect of spa therapy and short wave therapy in knee osteoarthritis: a randomized, single blind, controlled trial. Rheumatol Int.

[B18] Fransen M, Nairn L, Winstanley J, Lam P, Edmonds J (2007). Physical activity for osteoarthritis management: a randomized controlled clinical trial evaluating hydrotherapy or Tai Chi classes. Arthritis Rheum.

[B19] Garland D, Holt P, Harrington JT, Caldwell J, Zizic T, Cholewczynski J (2007). A 3-month, randomized, double-blind, placebo-controlled study to evaluate the safety and efficacy of a highly optimized, capacitively coupled, pulsed electrical stimulator in patients with osteoarthritis of the knee. Osteoarthritis Cartilage.

[B20] Herrero-Beaumont G, Ivorra JA, Del Carmen Trabado M, Blanco FJ, Benito P, Martin-Mola E (2007). Glucosamine sulfate in the treatment of knee osteoarthritis symptoms: a randomized, double-blind, placebo-controlled study using acetaminophen as a side comparator. Arthritis Rheum.

[B21] Karagulle M, Karagulle MZ, Karagulle O, Donmez A, Turan M (2007). A 10-day course of SPA therapy is beneficial for people with severe knee osteoarthritis: A 24-week randomised, controlled pilot study. Clin Rheumatol.

[B22] Kruger K, Klasser M, Mossinger J, Becker U (2007). Oxaceprol – a randomised, placebo-controlled clinical study in osteoarthritis with a non-conventional non-steroidal anti-inflammatory drug. Clin Exp Rheumatol.

[B23] Lambert RG, Hutchings EJ, Grace MG, Jhangri GS, Conner-Spady B, Maksymowych WP (2007). Steroid injection for osteoarthritis of the hip: a randomized, double-blind, placebo-controlled trial. Arthritis Rheum.

[B24] Louthrenoo W, Nilganuwong S, Aksaranugraha S, Asavatanabodee P, Saengnipanthkul S, Thai Study G (2007). The efficacy, safety and carry-over effect of diacerein in the treatment of painful knee osteoarthritis: a randomised, double-blind, NSAID-controlled study. Osteoarthritis Cartilage.

[B25] McCarthy G, O'Donovan J, Jones B, McAllister H, Seed M, Mooney C (2007). Randomised double-blind, positive-controlled trial to assess the efficacy of glucosamine/chondroitin sulfate for the treatment of dogs with osteoarthritis. Vet J.

[B26] Puopolo A, Boice JA, Fidelholtz JL, Littlejohn TW, Miranda P, Berrocal A (2007). A randomized placebo-controlled trial comparing the efficacy of etoricoxib 30 mg and ibuprofen 2400 mg for the treatment of patients with osteoarthritis. Osteoarthritis Cartilage.

[B27] Williamson L, Wyatt MR, Yein K, Melton JT (2007). Severe knee osteoarthritis: a randomized controlled trial of acupuncture, physiotherapy (supervised exercise) and standard management for patients awaiting knee replacement. Rheumatology (Oxford).

[B28] Yurtkuran M, Alp A, Konur S, Ozcakir S, Bingol U (2007). Laser acupuncture in knee osteoarthritis: a double-blind, randomized controlled study. Photomed Laser Surg.

[B29] Brandt KD (2001). A critique of the 2000 update of the American College of Rheumatology recommendations for management of hip and knee osteoarthritis. Arthritis Rheum.

[B30] Dieppe PA (2001). Concerns about the methodology used in developing the 2000 update of the American College of Rheumatology recommendations for management of hip and knee osteoarthritis. Arthritis Rheum.

[B31] Zhang W, Doherty M (2006). EULAR recommendations for knee and hip osteoarthritis: a critique of the methodology. Br J Sports Med.

[B32] Atkins D, Best D, Briss PA, Eccles M, Falck-Ytter Y, Flottorp S (2004). Grading quality of evidence and strength of recommendations. Bmj.

[B33] Atkins D, Briss PA, Eccles M, Flottorp S, Guyatt GH, Harbour RT (2005). Systems for grading the quality of evidence and the strength of recommendations II: pilot study of a new system. BMC Health Serv Res.

[B34] Blotman F, Maheu E, Wulwik A, Caspard H, Lopez A (1997). Efficacy and safety of avocado/soybean unsaponifiables in the treatment of symptomatic osteoarthritis of the knee and hip. A prospective, multicenter, three-month, randomized, double-blind, placebo-controlled trial. Rev Rhum Engl Ed.

[B35] Appelboom T, Schuermans J, Verbruggen G, Henrotin Y, Reginster JY (2001). Symptoms modifying effect of avocado/soybean unsaponifiables (ASU) in knee osteoarthritis. A double blind, prospective, placebo-controlled study. Scand J Rheumatol.

[B36] Maheu E, Mazieres B, Valat JP, Loyau G, Le Loet X, Bourgeois P (1998). Symptomatic efficacy of avocado/soybean unsaponifiables in the treatment of osteoarthritis of the knee and hip: a prospective, randomized, double-blind, placebo-controlled, multicenter clinical trial with a six-month treatment period and a two-month followup demonstrating a persistent effect. Arthritis Rheum.

[B37] Leeb BF, Schweitzer H, Montag K, Smolen JS (2000). A metaanalysis of chondroitin sulfate in the treatment of osteoarthritis. J Rheumatol.

[B38] McAlindon TE, LaValley MP, Gulin JP, Felson DT (2000). Glucosamine and chondroitin for treatment of osteoarthritis: a systematic quality assessment and meta-analysis. Jama.

[B39] Richy F, Bruyere O, Ethgen O, Cucherat M, Henrotin Y, Reginster JY (2003). Structural and symptomatic efficacy of glucosamine and chondroitin in knee osteoarthritis: a comprehensive meta-analysis. Arch Intern Med.

[B40] Reichenbach S, Sterchi R, Scherer M, Trelle S, Burgi E, Burgi U (2007). Meta-analysis: chondroitin for osteoarthritis of the knee or hip. Ann Intern Med.

[B41] Rintelen B, Neumann K, Leeb BF (2006). A meta-analysis of controlled clinical studies with diacerein in the treatment of osteoarthritis. Arch Intern Med.

[B42] Fidelix TS, Soares BG, Trevisani VF (2006). Diacerein for osteoarthritis. Cochrane Database Syst Rev.

[B43] Towheed TE, Maxwell L, Anastassiades TP, Shea B, Houpt J, Robinson V (2005). Glucosamine therapy for treating osteoarthritis. Cochrane Database Syst Rev.

[B44] Vlad SC, LaValley MP, McAlindon TE, Felson DT (2007). Glucosamine for pain in osteoarthritis: why do trial results differ?. Arthritis Rheum.

[B45] Reginster JY (2007). The efficacy of glucosamine sulfate in osteoarthritis: financial and nonfinancial conflict of interest. Arthritis Rheum.

[B46] Reichenbach S, Blank S, Rutjes AW, Shang A, King EA, Dieppe PA (2007). Hylan versus hyaluronic acid for osteoarthritis of the knee: a systematic review and meta-analysis. Arthritis Rheum.

[B47] Arrich J, Piribauer F, Mad P, Schmid D, Klaushofer K, Mullner M (2005). Intra-articular hyaluronic acid for the treatment of osteoarthritis of the knee: systematic review and meta-analysis. Cmaj.

[B48] Lo GH, LaValley M, McAlindon T, Felson DT (2003). Intra-articular hyaluronic acid in treatment of knee osteoarthritis: a meta-analysis. Jama.

[B49] Bellamy N, Campbell J, Robinson V, Gee T, Bourne R, Wells G (2006). Viscosupplementation for the treatment of osteoarthritis of the knee. Cochrane Database Syst Rev.

[B50] Wang CT, Lin J, Chang CJ, Lin YT, Hou SM (2004). Therapeutic effects of hyaluronic acid on osteoarthritis of the knee. A meta-analysis of randomized controlled trials. J Bone Joint Surg Am.

[B51] Modawal A, Ferrer M, Choi HK, Castle JA (2005). Hyaluronic acid injections relieve knee pain. J Fam Pract.

[B52] Medina JM, Thomas A, Denegar CR (2006). Knee osteoarthritis: should your patient opt for hyaluronic acid injection?. J Fam Pract.

[B53] Manicourt DH, Azria M, Mindeholm L, Thonar EJ, Devogelaer JP (2006). Oral salmon calcitonin reduces Lequesne's algofunctional index scores and decreases urinary and serum levels of biomarkers of joint metabolism in knee osteoarthritis. Arthritis Rheum.

[B54] Bingham CO, Buckland-Wright JC, Garnero P, Cohen SB, Dougados M, Adami S (2006). Risedronate decreases biochemical markers of cartilage degradation but does not decrease symptoms or slow radiographic progression in patients with medial compartment osteoarthritis of the knee: results of the two-year multinational knee osteoarthritis structural arthritis study. Arthritis Rheum.

[B55] Spector TD, Conaghan PG, Buckland-Wright JC, Garnero P, Cline GA, Beary JF (2005). Effect of risedronate on joint structure and symptoms of knee osteoarthritis: results of the BRISK randomized, controlled trial [ISRCTN01928173]. Arthritis Res Ther.

[B56] Bruyere O, Delferriere D, Roux C, Wark JD, Spector T, Devogelaer JP (2007). Effects of strontium ranelate on spinal osteoarthritis progression. Ann Rheum Dis.

[B57] Campbell J, Bellamy N, Gee T (2007). Differences between systematic reviews/meta-analyses of hyaluronic acid/hyaluronan/hylan in osteoarthritis of the knee. Osteoarthritis Cartilage.

[B58] Clegg DO, Reda DJ, Harris CL, Klein MA, O'Dell JR, Hooper MM (2006). Glucosamine, chondroitin sulfate, and the two in combination for painful knee osteoarthritis. N Engl J Med.

[B59] Rozendaal RM, Koes BW, van Osch GJ, Uitterlinden EJ, Garling EH, Willemsen SP (2008). Effect of glucosamine sulfate on hip osteoarthritis: a randomized trial. Ann Intern Med.

